# Diabetes surveillance – status and perspectives

**DOI:** 10.17886/RKI-GBE-2018-055

**Published:** 2018-05-16

**Authors:** Christian Schmidt, Christin Heidemann, Rebecca Paprott, Jens Baumert, Yong Du, Lars Gabrys, Thomas Ziese, Christa Scheidt-Nave

**Affiliations:** Robert Koch Institute, Berlin, Department of Epidemiology and Health Monitoring

Over the past few decades, Germany has seen a significant increase in the number of people diagnosed with diabetes mellitus. Treating the disease and the related long-term complications is now a central field of public health (PH) action. To meet this challenge, and taking diabetes as an example, the Robert Koch Institute (RKI) initiated a surveillance project in 2015. Accompanied by national and international experts and stakeholders of health reporting (GBE) at the federal state level, the project’s running time was set at four years [1].

Generally speaking, PH surveillance consists of continuously and systematically analysing health data from multiple sources to provide a rapid response in the form of health promoting measures [2].

The first step therefore consisted in developing adequate indicators. Based on a multitier Delphi procedure, these indicators were condensed to a set of core and additional indicators. In addition to epidemiologic parameters, these also contain indicators for risk factors and health care provision.

The data model to represent indicators is based both on RKI health monitoring data as well as on secondary data. The contributions presented in these proceedings on the current Health in Germany survey and the potential of Data Transparency Regulations highlights the potential for analysis in both data types.

Moreover, a telephone survey conducted by the RKI in 2017 as part of the diabetes surveillance will for the first time provide comprehensive representative data on the knowledge and information needs of people with and without diabetes in Germany.

A further focus will be on preparing the results specifically for individual target groups. This will build on the tested and developing new health reporting formats [3].

Diabetes, largely the consequence of unhealthy lifestyles and living conditions, at the same time plays a significant role in the development of additional diseases. Consequently, the aim is to apply the consensual indicators, data model, reporting formats and the accumulated expert knowledge to develop a PH surveillance for chronic diseases.
